# Exploring using HBsAg to predict interferon treatment course to achieve clinical cure in chronic hepatitis B patients: a clinical study

**DOI:** 10.3389/fimmu.2024.1528758

**Published:** 2025-01-10

**Authors:** Fei Yan, Fei Tang, Jing Chen, YiCheng Lin, XinYu Chen, Qin Du, WeiLi Yin, Jing Liang, Lei Liu, Fang Wang, BaiGuo Xu, Qing Ye, HuiLing Xiang

**Affiliations:** ^1^ The Third Central Clinical College of Tianjin Medical University, Tianjin Third Central Hospital, Tianjin Key Laboratory of Extracorporeal Life Support for Critical Diseases, Institute of Hepatobiliary Disease, Tianjin, China; ^2^ Department of Gastroenterology and Hepatology, Tianjin Third Central Hospital, Tianjin Key Laboratory of Extracorporeal Life Support for Critical Diseases, Institute of Hepatobiliary Disease, Tianjin, China; ^3^ Nankai University Affiliated Third Center Hospital, Tianjin Third Central Hospital, Tianjin Key Laboratory of Extracorporeal Life Support for Critical Diseases, Institute of Hepatobiliary Disease, Tianjin, China

**Keywords:** chronic hepatitis B (CHB), clinical cure, hepatitis b surface antigen (HBsAg), PEG-IFN α, model

## Abstract

**Objective:**

Although pegylated interferon α-2b (PEG-IFN α-2b) therapy for chronic hepatitis B has received increasing attention, determining the optimal treatment course remains challenging. This research aimed to develop an efficient model for predicting interferon (IFN) treatment course.

**Methods:**

Patients with chronic hepatitis B, undergoing PEG-IFN α-2b monotherapy or combined with NAs (Nucleoside Analogs), were recruited from January 2018 to December 2023 at Tianjin Third Central Hospital. All patients achieved hepatitis B surface antigen (HBsAg) clearance post-treatment.

**Result:**

The study enrolled 176 patients with chronic hepatitis B, with the median IFN treatment course of 35.23 ± 25.22 weeks. They were randomly divided into two cohorts in a ratio of 7:3. And there were 123 patients in the training cohort and 53 patients in the validation cohort. Univariable and multivariable analyses demonstrated that baseline HBsAg, 12 weeks HBsAg and the presence of cirrhosis significantly influenced IFN treatment course, and both are risk factors (β=7.27,4.27,10.91; p<0.05). After adjusting for confounding factors, HBsAg remained a significant predictor (β=6.99, 95%CI: 3.59,10.40; p<0.05), which was finally included to establish the model. The actual and predicted values in the validation cohort were highly matched, meanwhile the mean absolute percentage error (MAPE), root mean square error (RMSE) and accuracy (ACC) of the validation cohort were calculated. External validation also suggests that the model can be used as a tool for initial assessment.

**Conclusion:**

Baseline HBsAg in chronic hepatitis B patients were a risk factor for prolonged IFN treatment course with a positive correlation. Ultimately, a personalized model based on baseline HBsAg levels can be established to roughly estimate the duration of interferon therapy prior to treatment initiation, thereby guiding clinical decision-making.

## 1 Introduction

Recent World Health Organization (WHO) statistics (1) indicate that approximately 257 million individuals worldwide have been infected with HBV, with an additional 86 million cases of chronic HBV infection in China alone. According to the Chinese Medical Journal in 2022 (2, 3), in 2019, there were 162,000 HBV-related liver disease deaths in China. Among these, HBV-related cirrhosis and other chronic liver diseases comprised 26.04%, while liver cancer related to HBV constituted 72.18% of these fatalities. Recently, Professor Wen Juei Jeng of Taiwan Chang Gung Hospital and Professor Anna Lok, former President of the American Association for the Study of Liver Diseases (AASLD) (4), have emphasized that achieving clinical cure remains the optimal therapeutic goal for hepatitis B.

Functional cure, also known as clinical cure, for chronic hepatitis B (CHB) is characterized by sustained, undetectable serum HBsAg levels, and is associated with reduced risks of cirrhosis and hepatocellular carcinoma (HCC), and improved long-term prognosis. The addition of PEG-IFN α-2b to treatment for select patients has been shown effective for achieving clinical cure (5–8). Both domestic and international guidelines concur on a 48-week interferon treatment course for adult CHB patients, with extensions up to 72 weeks in some cases. Data from the Everest Project in China (9) revealed that for patients with HBsAg levels <1500 IU/ml, the HBsAg clearance rate at 48 weeks was 33.2%. However, the 48-week treatment course may not be sufficient for the majority of patients to achieve therapeutic goals. Notably, some patients with low HBsAg levels have achieved clinical cure within 24 weeks or less, underscoring the clinical significance of personalized estimation of the required treatment course for HBsAg clearance in CHB patients prior to initiation. Despite extensive research on factors influencing IFN treatment, few studies have explored the individualized course of IFN treatment. Therefore, this study aimed to develop a predictive model to guide clinicians in deciding when to discontinue interferon therapy for CHB patients.

## 2 Methods

### 2.1 Study design

This study adopted a single-center, retrospective design. Subjects were enrolled from January 2018 to December 2023 in the Departments of Gastroenterology and Hepatology at Tianjin Third Central Hospital. They received treatment with Pegylated interferon α-2b (PEG-IFN α-2b) either as monotherapy or in combination with nucleoside analogs (NAs). The primary endpoint was HBsAg clearance at the conclusion of treatment.

Inclusion Criteria: (i) Age 18 and 70 years; (ii) CHB or Hepatitis B-related compensated cirrhosis diagnosis (following the diagnostic criteria outlined in the Chinese Guidelines for the Prevention and Treatment of Chronic Hepatitis B [2022])(5); (iii) Received PEG-IFN α-2b therapy with HBsAg clearance during treatment; (iv) Not receiving immunosuppressive, hormonal medications. Exclusion Criteria: (i) Other chronic liver diseases, including viral hepatitis types A, C, D and E; (ii) Auto-immune hepatitis (AIH) or human immunodeficiency virus (HIV) infection; (iii) Interruptions in PEG-IFN α-2b treatment(for over 3 months); (iv) Insufficient essential data for analysis.

### 2.2 Observation indicators and concepts

Data collection encompassed basic patient information (including age, gender, previous treatment, and the treatment regimen for this course of therapy), initiation of PEG-IFN α-2b treatment, and time of HBsAg clearance (corresponding to the follow-up time for initial clearance of hepatitis B surface antigen). Baseline laboratory parameters: including hepatitis B surface antigen (HBsAg), hepatitis B e antigen (HBeAg), hepatitis B virus nucleic acid (HBV DNA); white blood cells (WBC), hemoglobin (Hb), platelets (PLT), alanine aminotransferase (ALT), total bilirubin (TBil), alkaline phosphatase (ALP), gamma-glutamyl transpeptidase (GGT), albumin (ALB). Dynamics parameters: including 12 weeks HBsAg, 24 weeks HBsAg, 12 weeks ALT. Imaging: abdominal ultrasound (liver, biliary, pancreas, spleen)

Baseline Time: initiation of PEG-IFN α-2b treatment; Primary Endpoint: achievement of clinical cure i.e., HBsAg clearance, defined as HBsAg <0.05 IU/mL. IFN Treatment course denotes the period from the initiation of PEG-IFN α-2b to the date of HBsAg clearance. Patients were advised to have regular assessments every 3 ± 1.5 months.

### 2.3 Laboratory tests and drugs

Hepatitis B virus markers assay: Detected by the Abbott ARCHITECT i4000SR system (Abbott Diagnostics, Abbott Park, IL, USA) and subjected to chemiluminescent microparticle immunoassay (CMIA) technology, using HBsAg Reagent Kit (6C36/08P08, Abbott, Ireland), Anti-HBs Reagent Kit (7C18/07P89, Abbott, Ireland), HBeAg Reagent Kit (6C32/07P64, Abbott GmbH, Germany), Anti-HBe Reagent Kit (6C34/07P63, Abbott GmbH, Germany). According to the manufacturer’s instructions, HBsAg<0.05IU/mL was negative; Anti-HBs≥10 mIU/mL was negative; HBeAg S/CO value <1.00 was negative; Anti-HBe S/CO value >1.00 was negative.

HBV DNA assay: There are usually two detection techniques, one is detected by the Anadas9850 platform for automated nucleic acid extraction and real-time polymerase chain reaction (PCR) system, using HBV DNA Reagent Kit (Amplly, China), covering a detection range of over 50 IU/mL. According to the manufacturer’s instructions, HBV DNA <50 IU/mL was negative. The other is automated sample processing using an AmpliPrep analyzer and automated nucleic acid amplification and detection using a TaqMan48 analyzer (Roche, Pleasanton, CA, United States), using HBV DNA Reagent Kit (Roche, USA) with a range of over 20 IU/mL. According to the manufacturer’s instructions, HBV DNA <20 IU/mL was negative.

Biochemical parameters and peripheral blood counts were performed by an automated biochemical analyzer and an automated blood cell analyzer (Wako Pure Chemical Industries, Ltd, Tokyo, Japan) for whole blood testing.

The medication used in the study, pegylated interferon α-2b (Pegbing), was administered at a dosage of 180 μg/dose or 135 μg/dose (Xiamen Amoytop Biotech Co., LTD, Xiamen, China).

### 2.4 Model prediction evaluation

In order to verify the feasibility of the model, it is necessary to compare the actual values with the predicted values, using the method of calculating the mean absolute percentage error (MAPE), root mean square error (RMSE), and the accuracy rate (ACC) of the model predicted values. MAPE: generally ranging from 0-10, the smaller the value the higher the accuracy; RMSE: reflecting the deviation of the predicted values from the actual values, the smaller the value, the better (10)


$$MAPE=\frac{1}{n}\sum_{t=1}^{n}|\frac{A_{t}-F_{t}}{A_{t}}|\times 100\%$$

$$RMSE=\sqrt{\frac{\sum_{t=1}^{n}{\left|A_{t}-F_{t}\right|}^{2}}{n}}$$

$$ACC=\frac{1}{n}\sum_{t=1}^{n}[1-\frac{\left|F_{t}-A_{t}\right|}{F_{t}}\times 100\%]$$

Note: At: actual value; Ft: predicted value

### 2.5 Statistical methods

Continuous variables are expressed as mean ± standard deviation (SD) or median (IQR: Q1, Q3). Categorical variables are presented as counts (percentages). Differences among continuous variables are assessed using the Student’s t-test or Kruskal-Wallis one-way analysis of variance (ANOVA). Categorical variables are compared using the chi-squared test or Fisher’s exact test as appropriate. Serum HBsAg and HBV DNA levels were logarithmically transformed for analysis. Bivariate correlations were assessed using Spearman’s rank correlation. Univariable and multiple linear regression were employed to analyze influencing factors. Indicators with significant independent correlations were identified, and a model was developed. Statistical significance was set at a two-tailed p-value < 0.05. All statistical analyses were conducted using the EmpowerStats (http://www.empowerstats.com, X&Y Solutions, Inc.). Graphs were created using GraphPad Prism version 10.3.1 software.

### 2.6 Ethics statement

This study strictly adhered to the ethical guidelines of the 1975 Declaration of Helsinki and was approved by the Ethics Committee of Tianjin Third Central Hospital (IRB2020-015-01). This is a retrospective, observational study, and it does not affect the patients’ treatment plan, progression or prognosis, and it meets the conditions of the informed consent waiver for patients.

## 3 Results

### 3.1 Flow diagram of patients enrolled in this study

**Figure 1:**
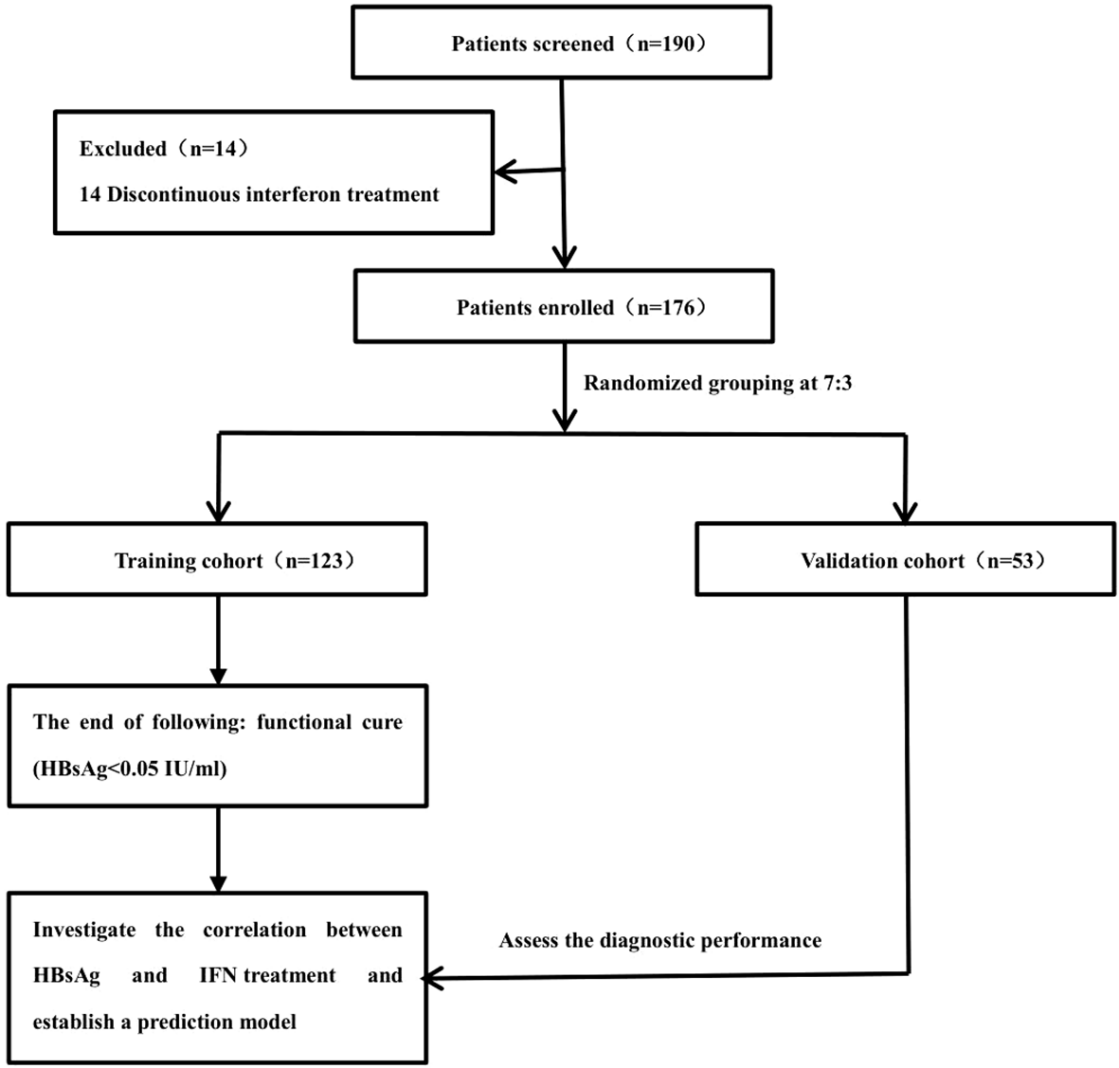
Flow diagram of patients enrolled in this study.

A total of 190 individuals were screened from the Department of Gastroenterology and Hepatology at Tianjin Third Central Hospital. And a total of 14 patients were excluded, all of whom had interruption of treatment during interferon therapy. Ultimately, 176 patients were included in the study. These patients were randomly divided into two cohorts at a 7:3 ratios, i.e., 123 subjects were assigned to the training cohort and the remaining 53 cases were assigned to the validation cohort. The primary endpoint was clinical cure during IFN therapy. This study aimed to investigate the correlation between HBsAg and IFN treatment, establish and validate a prediction model (**Figure 1**).

### 3.2 Characteristics of the study population

A total of 176 patients with CHB, with or without compensatory cirrhosis, were included in this study, comprising 118 males (67.05%), with an average age of 41.71 ± 8.84 years. The mean course of IFN treatment was 35.23 ± 25.22 weeks. Baseline HBsAg levels were 1.53 ± 1.33 log_10_ IU/ml. Among them, 13 patients were HBeAg-positive (7.39%). Out of 176 patients, only 132 had complete baseline data available, with 26.52% (35/132) diagnosed with cirrhosis. 96 patients (54.55%) had previously received interferon or nucleoside (acid) analogs. Among the 176 patients, 49 (27.84%) received IFN monotherapy, while 127 (72.16%) received IFN combined with NAs (ETV, TDF, TAF, and TMF).

Of 176 patients, 123 were training cohorts, with 87 males (70.73%) and an average age of 41.72 ± 9.21 years. The mean of IFN treatment was 34.32 ± 25.17 weeks. Baseline HBsAg levels were 42.22 (2.45-383.79) IU/ml, 53 were validation cohorts, with 31 males (58.49%) and an average age of 41.68 ± 8.01 years. The mean of IFN treatment was 26.00 (22.00-48.00) weeks. Baseline HBsAg levels were 51.87 (6.34-539.65) IU/ml. There was no significant difference of baseline characteristics between the training and validation cohorts except for the baseline ALB level (P=0.03) (**Table 1**).

**Table 1:** Clinical characteristics of the study population.

Characteristic	Overall (n = 176)	Training Cohort (n=123)	Validation Cohort (n=53)	P
Age/[y, Mean ± SD]	41.71 ± 8.84	41.72 ± 9.21	41.68 ± 8.01	0.98
Gender/n(%)				0.11
Male	118 (67.05%)	87 (70.73%)	31 (58.49%)	
Female	58 (32.95%)	36 (29.27%)	22 (41.51%)	
HBeAg/n(%)				0.25
(-)	163 (92.61%)	112 (91.06%)	51 (96.23%)	
(+)	13 (7.39%)	11 (8.94%)	2 (3.77%)	
Cirrhosis ^a^/n(%)				0.89
(-)	97 (73.48%)	63 (70.79%)	34 (79.07%)	
(+)	35 (26.52%)	26 (29.21%)	9 (20.93%)	
Previous treatment/n(%)				0.10
Naive	80 (45.45%)	50 (40.65%)	30 (56.60%)	
NAs treated	77 (43.75%)	57 (46.34%)	20 (37.74%)	
IFN treated	19 (10.80%)	16 (13.01%)	3 (5.66%)	
Treatment regimen/n(%)				0.66
IFN monotherapy	49 (27.84%)	34 (27.64%)	15 (28.30%)	
Combined with ETV	54 (30.68%)	40 (32.52%)	14 (26.42%)	
Combined with TDF	45 (25.57%)	29 (23.58%)	16 (30.19%)	
Combined with TAF	25 (14.20%)	17 (13.82%)	8 (15.09%)	
Combined with TMF	3 (1.70%)	3 (2.44%)	0 (0.00%)	
IFN treatment course ^b^/(week)				0.47
M (Q_1_, Q_3_)	24.50 (16.75-46.00)	24.00 (15.00-45.00)	26.00 (22.00-48.00)	
Mean ± SD	35.23 ± 25.22	34.32 ± 25.17	37.34 ± 25.45	
Baseline Data				
HBsAg/[IU/mL, M (Q_1_, Q_3_)]	45.38 (2.62-440.03)	42.22 (2.45-383.79)	51.87 (6.34-539.65)	0.34
HBsAg/[log_10_ IU/mL, Mean ± SD]	1.53 ± 1.33	1.49 ± 1.33	1.62 ± 1.34	0.56
HBV DNA/[log_10_ copies/ml, Mean ± SD]	1.06 ± 1.92	1.05 ± 1.99	1.07 ± 1.78	0.94
WBC/[10^9^/L, M (Q_1_, Q_3_)]	5.44 (4.74-6.65)	5.62 (4.66-6.47)	5.31 (4.82-6.70)	0.54
HB/[g/L, Mean ± SD]	149.28 ± 27.82	151.81 ± 28.68	145.54 ± 23.31	0.19
PLT/[10^9^/L, Mean ± SD]	215.03 ± 73.67	209.22 ± 70.25	225.48 ± 81.16	0.21
ALT/[U/L, M (Q_1_, Q_3_)]	21.00 (14.00-34.00)	23.00 (14.00-38.00)	20.50 (15.00-30.25)	0.22
TBiL/[umol/L, Mean ± SD]	17.76 ± 12.01	16.63 ± 7.24	19.36 ± 18.34	0.17
ALP/[U/L, Mean ± SD]	71.14 ± 25.02	71.56 ± 26.61	71.18 ± 21.75	0.93
GGT/[U/L, M (Q_1_, Q_3_)]	20.00 (13.75-28.00)	20.00 (13.00-28.25)	20.00 (14.00-27.00)	0.51
ALB/[g/L, Mean ± SD]	48.88 ± 4.55	49.34 ± 4.06	47.71 ± 5.33	0.03
Dynamic Data				
12w HBsAg/[log_10_ IU/mL, M (Q_1_, Q_3_)]	-0.15 (-1.51-1.50)	-0.33 (-1.51-1.44)	0.58 (-1.00-1.70)	0.13
HBsAg decline at 12 weeks/ [log_10_ IU/mL, M (Q_1_, Q_3_)]	1.17 (0.40-2.27)	1.35 (0.42-2.71)	0.99 (0.40-1.83)	0.28
24w HBsAg/[log_10_ IU/mL, M (Q_1_, Q_3_)]	-1.51 (-3.00-0.42)	-1.68 (-3.00-0.01)	-0.97 (-1.96-0.98)	0.13
HBsAg decline at 24 weeks/ [log_10_ IU/mL, M (Q_1_, Q_3_)]	2.57 (1.44-3.70)	2.73 (1.59-3.96)	2.20 (1.22-3.42)	0.22
12w ALT/[U/L, M (Q_1_, Q_3_)]	60.00 (38.00-90.00)	55.50 (38.00-85.00)	65.00 (36.50-100.00)	0.22
ALT raise at 12 weeks/[U/L, M (Q_1_, Q_3_)]	30.00 (11.00-60.00)	29.00 (11.00-55.00)	36.00 (11.50-76.00)	0.58

SD, standard deviation; M, Median; Q_1_, 1st Quartile; Q_3_, 3st Quartile; CHB, Chronic hepatitis B; PEG-IFN α-2b, Pegylated interferon α-2b; NAs, Nucleoside Analogs; ETV, Entecavir; TDF, Tenofovir disoproxil fumarate; TAF, Tenofovir alafenamide fumarate tablets; TMF, Tenofovir amibufenamide; HBsAg, hepatitis B surface antigen; HBeAg, hepatitis B envelope antigen; HBV DNA, hepatitis B virus deoxyribonucleic acid; WBC, White Blood Cell; HB, Hemoglobin; PLT, Platelet; ALT, Alanine aminotransferase; TBil, total bilirubin; ALP, Alkaline Phosphatase; GGT, Gamma-glutamyltransferase; ALB, albumin.

The data are presented as means ± standard deviation; skewed data are presented as the median (IQR), and categorical data as the number (percentage).

a Out of 176 patients, only 132 had complete baseline data available, with 26.52% (35/132) diagnosed with cirrhosis. 29.21% (26/89) of patients in the training cohort had cirrhosis and 20.93%(9/43) in the validation cohort had cirrhosis.

b Represents the period from the initiation of Peg-IFNα-2b treatment to the HBsAg clearance.

### 3.3 Univariable and multiple regression analysis of variables with IFN treatment

Using clinical cure as the study endpoint, univariable and multiple regression analyses were conducted on baseline and dynamic indicators. Univariable analysis revealed that baseline, 12 and 24 weeks HBsAg, baseline HBeAg and the presence of cirrhosis all significantly impacted IFN treatment (P < 0.05). Multiple regression analysis indicated that baseline, 12 weeks HBsAg and the presence of cirrhosis were significant factors affecting the course of IFN treatment, and both were risk factors (β=7.27,4.27,10.91; p<0.05), i.e., the smaller the baseline and 12 weeks HBsAg in non-cirrhotic patients, the better the response to interferon, and the shorter the course of treatment to achieve clinical cure. After adjusting for confounding factor which is gender, age, 12 weeks HBsAg and the presence of cirrhosis, it was found that baseline HBsAg level remained a predictor of IFN treatment (β=6.99, 95%CI: 3.59,10.40; p<0.05) (**Tables 2**, **3**).

**Table 2:** Uni-variate and multiple linear regression analysis of factors associated with IFN treatment course.

Variables	Uni-variable		Multi- variable	
	β (95%CI)	P	β (95%CI)	P
Age/y	-0.39 (-0.87, 0.10)	0.12		
Gender				
Male	0			
Female	-3.63 (-13.43, 6.17)	0.47		
HBeAg				
(-)	0		0	
(+)	23.11 (8.01, 38.22)	<0.01	9.88 (-5.91, 25.67)	0.22
Cirrhosis				
(-)	0		0	
(+)	13.66 (3.08, 24.24)	0.01	10.91 (1.74, 20.07)	0.02
Previous treatment				
Naive	0			
NAs treated	8.49 (-0.98, 17.97)	0.08		
IFN treated	-2.45 (-16.50, 11.60)	0.73		
Treatment regimen				
IFN monotherapy	0			
Combined with ETV	8.39 (-2.95, 19.73)	0.15		
Combined with TDF	11.67 (-0.62, 23.95)	0.07		
Combined with TAF	-4.76 (-19.21, 9.68)	0.52		
Combined with TMF	17.94 (-11.34, 47.22)	0.23		
Baseline Data				
HBsAg/(log_10_ IU/mL)	10.31 (7.48, 13.13)	<0.01	7.27 (3.68, 10.86)	<0.01
HBV DNA/(log_10_ copies/ml)	1.57 (-0.68, 3.81)	0.17		
WBC/(10^9^/L)	0.06 (-0.08, 0.20)	0.38		
HB/(g/L)	0.01 (-0.16, 0.18)	0.90		
PLT/(10^9^/L)	-0.03 (-0.10, 0.03)	0.33		
ALT/(U/L)	-0.01 (-0.11, 0.08)	0.81		
TBiL/(umol/L)	-0.18 (-0.83, 0.47)	0.58		
ALP/(U/L)	-0.03 (-0.21, 0.15)	0.74		
GGT/(U/L)	-0.02 (-0.07, 0.02)	0.28		
ALB/(g/L)	0.18 (-0.98, 1.34)	0.76		
Dynamic Data				
12w HBsAg/(log_10_ IU/mL)	6.73 (4.50, 8.97)	<0.01	4.27 (1.25, 7.30)	<0.01
HBsAg decline at 12 weeks/(log_10_ IU/mL)	-1.45 (-4.17, 1.26)	0.297		
24w HBsAg/(log_10_ IU/mL)	5.64 (3.21, 8.07)	<0.01	0.76 (-2.54, 4.06)	0.65
HBsAg decline at 24 weeks/(log_10_ IU/mL)	0.08 (-2.45, 2.62)	0.949		
12w ALT/(U/L)	-0.05 (-0.15, 0.05)	0.36		
ALT raise at 12 weeks/(U/L)	-0.01 (-0.09, 0.06)	0.716		

PEG-IFN α-2b, Pegylated interferon α-2b; NAs, Nucleoside Analogs; ETV, Entecavir; TDF, Tenofovir disoproxil fumarate; TAF, Tenofovir alafenamide fumarate tablets; TMF, Tenofovir amibufenamide; HBsAg, hepatitis B surface antigen; HBeAg, hepatitis B envelope antigen; HBV DNA, hepatitis B virus deoxyribonucleic acid; WBC, White Blood Cell; HB, Hemoglobin; PLT, Platelet; ALT, Alanine aminotransferase; TBil, total bilirubin; ALP, Alkaline Phosphatase; GGT, Gamma-glutamyl transferase; ALB, albumin.

IFN treatment course: the period from the initiation of Peg-IFNα-2b treatment to the HBsAg clearance.

p<0.05 is considered significant difference.

**Table 3:** Analysis of the effect of HBsAg on IFN treatment course after adjusting confounding factor.

Variables	Model 1		Model 2		Model 3	
	β (95%CI)	P	β (95%CI)	P	β (95%CI)	P
Baseline HBsAg (log_10_ IU/mL)	10.31 (7.48 13.13)	<0.01	10.29 (7.37 13.20)	<0.01	6.99 (3.59 ~ 10.40)	<0.01

CI, Confidence Interval.

Model 1: Only Variables HBsAg; Model 2: Adjust: Gender; Age; Model 3: Gender; Age; 12wHBsAg; Cirrhosis.

### 3.4 Curve fitting and predicted model between HBsAg and IFN treatment

**Figure 2:**
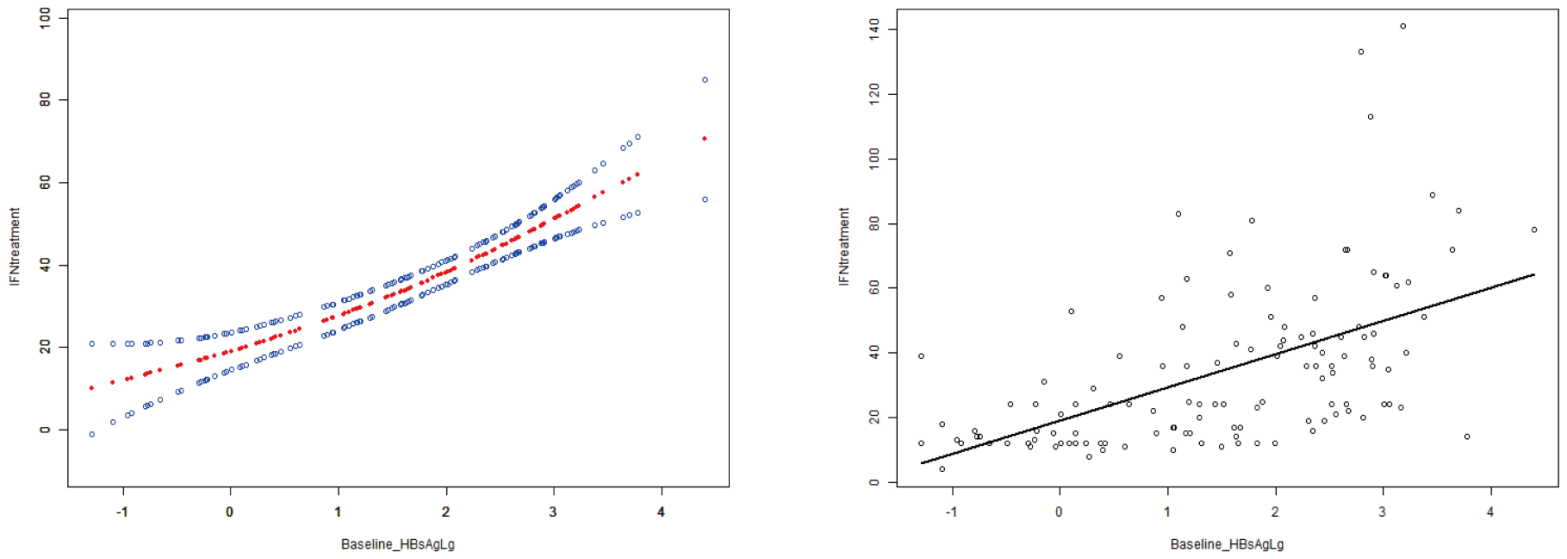
The curve fitting and correlation analysis between IFN treatment course and baseline HBsAg. Spearman correlation analysis indicated that there was a significant positive correlation between the two variables (rs=0.61,p<0.001).

**Figure 3:**
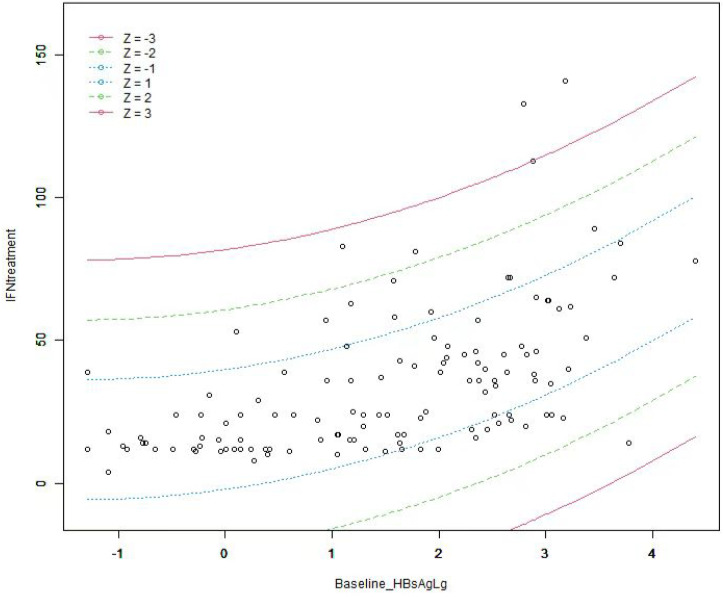
The distribution of IFN treatment course under different baseline HBsAg. The term Z represents the standard deviation (SD). Individuals outside the red line are abnormal(outliers) value, and the people within 3 times SD cover 99% of the population, indicating that the fitting effect of this model is ideal.

Curve fitting and Spearman correlation analysis of baseline HBsAg and IFN treatment course revealed a significant positive association between the two variables (r_s_ = 0.61, P < 0.05). HBsAg was finally incorporated to establish a prediction model, Y = 21.83 + 5.25*X + 1.93*X^2^, Y: IFN treatment, X: Log (baseline HBsAg), with an R^2^ value of 0.32, which is a better fitting efficacy than the linear model(R^2^ = 0.29). The distribution of IFN treatment course corresponding to different HBsAg showed 3 standard deviations covering 99% of the population, with only a few individuals located outside the red line (i.e., outliers), indicating an ideal fitting effect of this model (**Figures 2**, **3**).

### 3.5 Internal validation of the predictive ability of models

**Figure 4:**
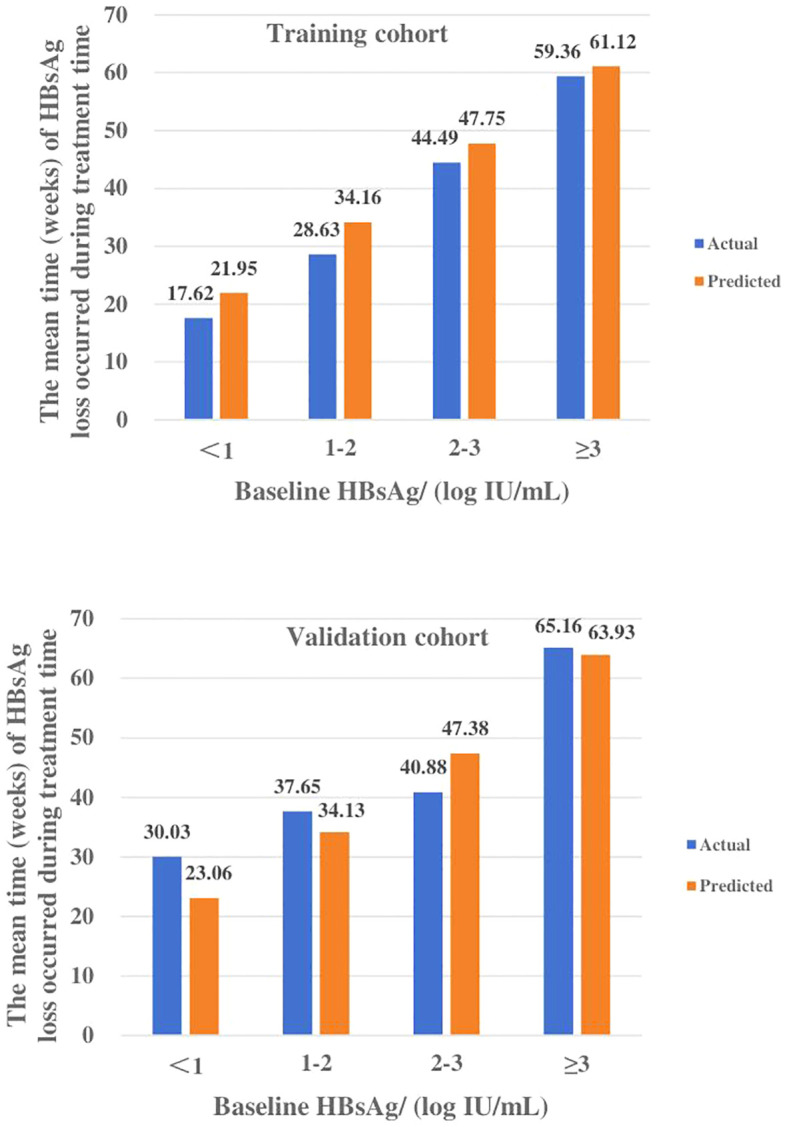
The median time (weeks) of HBsAg loss under different baseline HBsAg levels in the training and validation cohort. Actual and predicted values of IFN treatment course for each subgroup of the training and validation cohorts were highly compatible.

The patients were divided into four groups based on baseline HBsAg levels, which were <1, 1-2, 2-3, and ≥3 log_10_ IU/ml. The actual values of IFN treatment course for these groups in the training cohort were 17.62 ± 11.39, 28.63 ± 20.26, 44.49 ± 25.50, and 59.36 ± 32.55 weeks, and 30.03 ± 25.76, 37.65 ± 19.64, 40.88 ± 31.20 and 65.16 ± 25.13 weeks for the validation cohort, respectively (**Table 4**, **Figure 4**).

**Table 4:** IFN treatment course under different baseline HBsAg groups in a Training and Validation Cohort.

Variables	IFN treatment course (Mean ± SD)			
	Training Cohort		Validation Cohort	
	Actual value	Predicted value	Actual value	Predicted value
<1*	17.62 ± 11.39	21.95 ± 3.04	30.03 ± 25.76	23.06 ± 3.37
1-2*	28.63 ± 20.26	34.16 ± 3.39	37.65 ± 19.64	34.13 ± 2.97
2-3*	44.49 ± 25.50	47.75 ± 3.97	40.88 ± 31.20	47.38 ± 5.17
≥3*	59.36 ± 32.55	61.12 ± 7.29	65.16 ± 25.13	63.93 ± 10.82
MAPE/%	0.14		0.13	
RMSE	3.97		5.12	
ACC/%	88.59		85.95	

SD, standard deviation; HBsAg, Hepatitis B surface antigen; *Grouping, four groups by baseline HBsAg level (log_10_ IU/ml); MAPE, Mean absolute percentage error, generally between 0-10, the smaller the value the higher the accuracy; RMSE, Root mean squared error, the smaller the better; ACC, Accuracy.

The predicted values of IFN treatment course calculated by the model were also divided into four subgroups and compared with the actual values. The means of each subgroup of the two sets of data were highly matched. In the training cohort, the MAPE and RMSE were 0.14, 3.97, respectively and its ACC is 88.59%, while MAPE and RMSE in the validation cohort are 0.13 and 5.12, respectively, and ACC is 85.95%, which is a good degree of fit (**Table 4**).

## 4 Discussion

CHB stands as one of the most severe infections, contributing to an estimated 73% of all cancer-related deaths globally (11). The treatment goal revolves around long-term inhibition of HBV replication to delay and mitigate complications, with clinical cure being pursued for select patients under specific conditions (5). Prior research has shown that HBsAg clearance significantly reduces the incidence of complications like cirrhosis and hepatocellular carcinoma (12, 13). However, the annual spontaneous clearance rate of HBsAg stands at approximately 1.17% (14). While nucleoside analogs (NAs) effectively suppress viral replication, clinical cure remains rare, with an annual incidence of merely 0.22% (15, 16). In contrast, interferon treatment exhibits a higher antiviral response rate, facilitating clinical cure in more CHB patients (17). This is attributed to chronic HBV infection inducing immune tolerance, and interferon’s dual antiviral and immune-regulatory effects contribute to a higher clinical cure rate in CHB treatment (18).

A meta-analysis based on 7913 patients and relevant literature (16, 19) highlighted that younger age, female gender, high ALT levels, and low HBsAg levels—termed the advantaged population—exhibit the best-sustained response to PEG-IFN α and a higher virus response rate. Recent retrospective studies and subgroup analyses of OSST trials (20–23) further revealed that patients with low baseline HBsAg levels are more likely to achieve HBsAg clearance. Compared to those with HBsAg levels >1500 IU/ml, patients with HBsAg <1500 IU/ml at baseline showed significantly enhanced clearance rates (22.2% *vs*. 1.7%). Besides baseline characteristics, Hou Jinlin’s studies (24) found that HBsAg levels in different treatment periods can also affect the course of interferon therapy, multiple studies (25, 26) have also demonstrated that dynamic data can correlate with interferon treatment outcomes. Patients with a rapid decline in HBsAg during the initial 12 weeks of treatment (77.2% *vs*. 29.2%, P < 0.05) were more likely to achieve HBsAg clearance within 48 weeks. Conversely, a slow decline necessitated treatment prolongation beyond 48 weeks to attain clinical cure. Baseline HBsAg levels, as well as levels during treatment, play pivotal roles in determining clinical cure. In this study, we show that baseline, 12 weeks HBsAg and the presence of cirrhosis were significant factors affecting the course of IFN treatment, and both were risk factors (β=7.27,4.27,10.91; p<0.05). Spearman’s analysis revealed that there was a significant positive correlation between them (r_s_=0.61, P<0.05). The above statistical results also accord with our earlier observations, suggesting that it is feasible to predict IFN treatment by baseline HBsAg.

There is a scarcity of studies regarding the prediction of IFN treatment course. Currently, domestic and international guidelines recommend a 48-week IFN treatment regimen (5–7). However, data from the China Everest Project and studies by Han Meifang, among others (9, 21, 26), have shown that only 33.2%/51.4% of patients achieved HBsAg clearance within 48 weeks, necessitating extended treatment for other patients. Despite extending the treatment course from 48 weeks to 96 weeks, the HBsAg clearance rate increased by only 7.3%. Li et al. (27) identified that 14.7% of patients with low baseline HBsAg achieved clinical cure within 24 weeks. The recommended treatment course of 48 or 96 weeks may not be suitable for most CHB patients. Furthermore, interferon therapy is associated with more adverse reactions and higher costs compared to NAs, contributing to patient reluctance. Therefore, developing an IFN treatment prediction model before treatment holds significant importance in enhancing the acceptance of interferon therapy among doctors and patients, ultimately enabling more patients to achieve clinical cure. Existing studies (16, 19–23) have reported the correlation between HBsAg and interferon treatment, but a specific quantitative relationship was lacking. Thus, a predictive model for interferon treatment duration is constructed to estimate the anticipated course and cost prior to the commencement of therapy. Independent influencing factors were screened using uni- and multi-variables analysis, and the baseline HBsAg ultimately is incorporated to establish a prediction model. The predicted values were compared with the actual values, and in the validation cohort, the MAPE and RMSE were 0.13 and 5.12, respectively, ACC was 85.95%, which was a good degree of fit. In addition, Li et al. (27) also concluded that patients with lower baseline HBsAg levels were more likely to achieve HBsAg loss, and the median time to HBsAg clearance for patients in the baseline HBsAg < 1, 1-2, 2-3, 3-4, and ≥4 log IU/mL groups was 26.5, 39, 54.6, 68, and 65.7 weeks, which is in general accordance with our results, with a total of four subgroups falling within the predictive range (**Table 5**).

**Table 5:** The IFN treatment course of publicly data and prediction group.

**Baseline HBsAg/(log_10_ IU/mL)**	<1	1-2	2-3	3-4	≥4
**publicly data group(week)**	26.5	39	54.6	68	65.7
**prediction group(week)**	<29.01	29.01-40.05	40.05-54.95	54.95-73.71	≥73.71

HBsAg, Hepatitis B surface antigen. “Public data group (week)": the median time to HBsAg clearance for patients in the different baseline HBsAg groups as demonstrated in the study by Li et al. (27). "prediction group(week)": the predicted time to HBsAg clearance calculated by the prediction model which we established.

The innovation of this study lies in providing a quantitative model for forecasting IFN treatment course, which is of great significance for clinicians to accurately grasp realistic factors such as therapy cost before treatment. However, this is a single-center retrospective study with an insufficient sample size, potentially leading to biased results. Therefore, we are conducting a multicenter study to expand the sample size, which has also concluded that baseline HBsAg levels are factors that significantly influence IFN treatment, but the exact results have not yet been published. Additionally, HBsAg and ALT levels during treatment are also key factors, and the factors incorporated into the model can be further improved. But the primary aim of our study is to assist clinicians in preliminarily estimating the duration of interferon therapy before treatment, which is also one of the concerns for most patients. Despite these limitations, the establishment of this prediction model realizes the personalized interferon treatment for patients with chronic hepatitis B. It can guide clinicians’ decisions to a certain extent and lay the foundation for further research.

In summary, baseline HBsAg in chronic hepatitis B patients were a risk factor for prolonged IFN treatment course with a positive correlation. Ultimately, a personalized model based on baseline HBsAg levels can be established to roughly estimate the duration of interferon therapy prior to treatment initiation, thereby guiding clinical decision-making.

## Data Availability

The raw data supporting the conclusions of this article will be made available by the authors, without undue reservation.
